# Enhancing SSVEP-Based Brain-Computer Interface with Two-Step Task-Related Component Analysis

**DOI:** 10.3390/s21041315

**Published:** 2021-02-12

**Authors:** Hyeon Kyu Lee, Young-Seok Choi

**Affiliations:** Department of Electronics and Communication Engineering, Kwangwoon University, Seoul 01897, Korea; skgusrb12@kw.ac.kr

**Keywords:** brain-computer interface (BCI), electroencephalography (EEG), steady-state visual evoked potential (SSVEP), canonical correlation analysis (CCA), task-related component analysis (TRCA), two-step task-related component analysis (TSTRCA)

## Abstract

Among various methods for frequency recognition of the steady-state visual evoked potential (SSVEP)-based brain-computer interface (BCI) study, a task-related component analysis (TRCA), which extracts discriminative spatial filters for classifying electroencephalogram (EEG) signals, has gathered much interest. The TRCA-based SSVEP method yields lower computational cost and higher classification performance compared to existing SSVEP methods. In spite of its utility, the TRCA-based SSVEP method still suffers from the degradation of the frequency recognition rate in cases where EEG signals with a short length window are used. To address this issue, here, we propose an improved strategy for decoding SSVEPs, which is insensitive to a window length by carrying out two-step TRCA. The proposed method reuses the spatial filters corresponding to target frequencies generated by the TRCA. Followingly, the proposed method accentuates features for target frequencies by correlating individual template and test data. For the evaluation of the performance of the proposed method, we used a benchmark dataset with 35 subjects and confirmed significantly improved performance comparing with other existing SSVEP methods. These results imply the suitability as an efficient frequency recognition strategy for SSVEP-based BCI applications.

## 1. Introduction

The brain-computer interface (BCI) provides a bidirectional system between the human brain and external devices by decoding electrical brain waves measured in specific environments. Among various measurements of brain activities, electroencephalography (EEG) is the most common tool in BCI systems due to inexpensive cost, portability, usability, and so forth [1]. EEG-based BCI may help severely disabled people, which is used in rehabilitative applications and the internet of medical things (IoMT) [2,3]. Typically, in years past, real-time BCI applications such as brain-controlled vehicles (BCVs) [4] and brain-controlled wheelchairs (BCWs) [5] that can be facilitated in daily life have received enormous attention. To control these applications, in the BCI study, EEG signals can be divided into different forms depending on the purpose of use, its type, and so on. Among those forms, steady-state visual evoked potential (SSVEP) has attracted much attention due to the high communication rate, classification accuracy, and high signal-to-noise ratio (SNR) [6,7]. Driven by these advantages, the number of SSVEP-based real-time BCI applications have resulted in remarkable achievements [4,5,8,9].

In SSVEP-based BCIs, in terms of detection target frequency among visual stimuli with specific frequencies and spatial filtering techniques have been widely developed due to high SNR by removing the external noise of EEG signals caused by artifacts and eye blinks. Recently, canonical correlation analysis (CCA) has been presented to identify target frequency based on the use of sinusoidal signals as reference signals [10]. Due to its high efficiency and easy implementation, CCA has been widely utilized in SSVEP-based BCI research. Moreover, according to other studies [11,12], CCA with a high information transfer rate (ITR) has shown great potential in online BCI applications. However, due to the interference in spontaneous EEG signals, CCA may suffer from its degradation of detection performance. To address this issue, in much of the literature, a number of variants of CCA have been proposed to achieve higher frequency recognition performance. For example, individual CCA (IT-CCA) [12], L1-regularized multi-way CCA (L1-MCCA) [13], multiset CCA (MsetCCA) [14], and latent common source extraction (LCSE) [15] have gained interest among BCI communities. Among those, the combination of the standard CCA and IT-CCA has led to the highest performance [16].

As another approach, several spatial filtering methods have shed light on frequency recognition to extract task-specific source activities from EEG signals. Among them, the task-related component analysis (TRCA) [17] has been developed to extract the spatial filter closely related to task-specific by finding the maximum correlation of the internal component between trials. Based on this approach, Nakanishi et al. introduced TRCA into the SSVEP-based BCI, leading to the best performance among existing methods [18]. The TRCA method achieved ITR of 325.33 ± 38.17 bits/min, implying practicality in real-life BCI application. More recently, an ensemble approach for incorporating a generated spatial filter has shown significant improvement to frequency recognition regardless of time window length [16]. In this line of thought, fusing all the canonical correlation coefficient of CCA yielded robust results and improved performance in terms of classification accuracy and ITR compared to CCA [19]. However, in spite of several advantages of the aforementioned methods, SSVEP-based BCI still suffers from the degradation of performance in cases where a short time window (TW) of EEG signals is used. In order to be available in online SSVEP-based BCI applications, robustness regarding the TW is an essential property of frequency recognition in SSVEP.

In this study, we present a novel frequency recognition method for SSVEP-based BCI by expanding the concept of the standard TRCA. The proposed method consists of two subsequent steps, which is referred to as a two-step TRCA (TSTRCA). First, we generate the subject-specific spatial filter using the standard TRCA. Then, motivated by an ensemble approach, the target frequency recognition is carried out by ensembling and emphasizing discriminative information from the correlation between the individual templates and test data. Thus, the proposed TSTRCA method can improve performance in a short TW by reflecting the correlation of inter-subjects as well as inter-sessions and accentuating features as ensemble classifiers. We validated the frequency recognition performance of the proposed method using the SSVEP benchmark dataset, comprised of 35 subjects [20]. In addition, we compared the average accuracy and ITR of the proposed TSTRCA with CCA, extended CCA (ExtCCA), and TRCA.

The rest of this paper is organized as follows: Section 2 presents an introduction of the benchmark SSVEP dataset and describes existing methods and the proposed method. In Section 3, the experimental results are exhibited. Section 4 provides the conclusion of this study.

## 2. Materials and Algorithms

### 2.1. Benchmark SSVEP EEG Dataset and Preprocessing

In this study, the benchmark SSVEP dataset provided by Wang et al. [20] was utilized to evaluate the proposed method. Thirty-five healthy subjects, consisting of seventeen females and eighteen males, participated in an SSVEP experiment by staring at an offline 40-target BCI speller (5 × 8 character matrix), each with a different frequency. The 40-target BCI speller has a range between 8 Hz and 15.8 Hz with an interval of 0.2 Hz. The SSVEP EEG signals were recorded with 64 channels, sampled at 1000 Hz, and band-pass filtered between 0.15 Hz and 200 Hz. A notch filter at 50 Hz was employed in order to remove power-line interference. For each subject, the dataset was made up of a total of 6 s per trial, and one trial was repeated six times. In each trial, a visual cue indicating the beginning of the experiment appeared for 0.5 s.

All 40 target frequencies were presented randomly to all subjects. After the end of the visual stimulation, it was blanked for 0.5 s before the next experiment was presented. During the experiment, the subject was asked to avoid blinking and a suitable rest was also provided between two consecutive trials.

To facilitate the signal-processing analytics, the SSVEP datasets were further preprocessed. Firstly, the band-pass filter with an IIR filter was applied to all data epochs. The frequency range was considered from 7 Hz to 90 Hz. Then, as shown in [20], considering a latency delay in the visual system, the SSVEP data were extracted between 0.64 s and 0.64 + ds from each epoch, where ds is the length of TW for frequency recognition. The supplementary information for this dataset was elucidated in [20].

### 2.2. Conventional SSVEP Frequency Recognition Methods

In this section, we first provide a brief introduction of the conventional SSVEP frequency recognition methods such as CCA, ExtCCA, and TRCA, which is followed by the proposed method. Then, the frequency recognition using a filter bank approach, which is known as its capability to improve the performance of standard SSVEP methods, is provided. 

#### 2.2.1. Standard Canonical Correlation Analysis

CCA is a conventional statistical method to explore the underlying correlation between two sets of multidimensional variables. Assume that multidimensional signals are given as $X\in {\mathbb{R}}^{D_{1}\times T}$ and $Y\in {\mathbb{R}}^{D_{2}\times T}$; CCA aims at finding a pair of weight vectors, $w_{x}\in {\mathbb{R}}^{D_{1}\times 1}$ and $w_{y}\in {\mathbb{R}}^{D_{2}\times 1}$, which maximize the correlation between their linear combinations $x=w_{x}{}^{T}X$ and $y=w_{y}{}^{T}Y$. Formally, the correlation coefficient of CCA is given by

(1) $$\rho =\;\frac{E\left[xy^{T}\right]}{\sqrt{E\left[xx^{T}\right]E\left[yy^{T}\right]}}\;\;\;\;\;\;\;=\;\frac{w_{x}{}^{T}XY^{T}w_{y}}{\sqrt{w_{x}{}^{T}XX^{T}w_{x}}\sqrt{w_{y}{}^{T}YY^{T}w_{y}}}$$

where ρ is the Pearson correlation coefficient between x and y. Then, the weight vectors $w_{x}$ and $w_{y}$ based on CCA is obtained by maximizing the correlation ρ in Equation (1). Formally, this problem for finding the weight vectors $w_{x}$ and $w_{y}$ can be represented by

(2) $$\arg\underset{w_{x},\;w_{y}}{\;\max}\;\rho =\;w_{x}{}^{T}XY^{T}w_{y}\;s.t.\;w_{x}{}^{T}XX^{T}w_{x}=1,w_{y}{}^{T}YY^{T}w_{y}=1$$

Then, the optimal weight vectors are obtained through a generalized eigenvalue problem [21]. Here, the maximum of ρ regarding the weight vectors is referred to as the maximum canonical correlation. 

In the SSVEP-based BCI, CCA has been widely used for frequency recognition by obtaining the maximum canonical correlation between test signals of SSVEP EEGs and reference signals [10]. In addition, the reference signals are composed of sinusoidal signals, denoted as $Z_{i}\in {\mathbb{R}}^{2N_{h}\times N_{s}},\;i=1,\;2,\;\cdots ,\;N_{f}$, which is given by

(3) $$Z_{i}=\;\left(\begin{array}{c} \sin\left(2\pi f_{i}t\right)\\ \cos\left(2\pi f_{i}t\right)\\ \ldots \\ \sin\left(2\pi N_{h}f_{i}t\right)\\ \cos\left(2\pi N_{h}f_{i}t\right) \end{array}\right),\;\;t=\;\frac{1}{F_{s}},\;\frac{2}{F_{s}},\;\ldots ,\;\frac{N_{s}}{F_{s}}$$

where, $N_{h}$ is the number of harmonics, $N_{s}$ is the number of sample points, $N_{f}$ is the number of target frequencies, and $F_{s}$ is the sampling rate. However, for the reference signals, it is difficult to determine the appropriate number of harmonics. Therefore, in the current study, we employed the individual template proposed by IT-CCA as the reference signals [12]. The test signal consists of a single trial of multichannel EEG signals, written as $\bar{X}\in {\mathbb{R}}^{N_{c}\times N_{s}}$, where $N_{c}$ denotes the number of channels. 

Finally, the target frequency is identified in the cases where the correlation coefficient $\rho_{i}$—calculated by CCA between a test signal and each reference signal—is at its maximum, as follows:

(4) $$f_{target}=\;\underset{i}{\max}\rho_{i},\;\;i=\;1,\;2,\;\cdots ,\;N_{f}$$

#### 2.2.2. Extended Canonical Correlation Analysis 

In [12,22], ExtCCA enhanced the signal to noise (SNR) of SSVEP by combining two frequency recognition methods, i.e., the standard CCA and IT-CCA. IT-CCA is a variant of the standard CCA in that the individual templates are used as the reference signals. The individual template, denoted as $Y_{i}\in {\mathbb{R}}^{N_{c}\times N_{s}},i=1,\;2,\;\cdots ,\;N_{f}$, is constructed by averaging across multiple EEG trials acquired from the same subjects. Furthermore, ExtCCA makes use of three weight vectors generated by three kinds of EEG signals, i.e., test signal and two reference signals, as the spatial filters. Specifically, three spatial filters are as follows: (1) $W_{\bar{X}}\left(\bar{X}Y_{i}\right)$ between the test signal $\bar{X}$ and the individual template $Y_{i}$, (2) $W_{\bar{X}}\left(\bar{X}Z_{i}\right)$ between the test signal $\bar{X}$ and a set of sinusoidal signals $Z_{i}$, (3) $W_{\bar{X}}\left(Y_{i}Z_{i}\right)$ between the individual template and a set of sinusoidal signals. A correlation vector $r_{i}\;,\;i=1,\;2,\;\cdots ,\;N_{f}$, for the i-th template signal can be obtained using the designed spatial filters as follows:

(5) $$r_{i}=\;\left[\begin{array}{c} r_{i,\;1}\\ r_{i,\;2}\\ r_{i,\;3}\\ r_{i,\;4} \end{array}\right]=\left[\begin{array}{c} \rho \left({\bar{X}}^{T}W_{\bar{X}}\left(\bar{X}Z_{i}\right),Z_{i}^{T}W_{Z}\left(\bar{X}Z_{i}\right)\right)\\ \rho \left({\bar{X}}^{T}W_{\bar{X}}\left(\bar{X}Y_{i}\right),Y_{i}^{T}W_{\bar{X}}\left(\bar{X}Y_{i}\right)\right)\\ \begin{array}{c} \rho \left({\bar{X}}^{T}W_{\bar{X}}\left(\bar{X}Z_{i}\right),Y_{i}^{T}W_{\bar{X}}\left(\bar{X}Z_{i}\right)\right)\\ \rho \left({\bar{X}}^{T}W_{\bar{X}}\left(Y_{i}Z_{i}\right),Y_{i}^{T}W_{\bar{X}}\left(Y_{i}Z_{i}\right)\right) \end{array} \end{array}\right]$$

where $\rho \left(\cdot \;,\;\cdot \right)$ is the Pearson correlation coefficient between two multidimensional SSVEP EEG signals. For each target frequency, the four correlation values in Equation (5) are combined as a weighted correlation coefficient $\rho_{i}$, which is given by

(6) $$\rho_{i}=\;\overset{4}{\underset{k=1}{\sum }}\mathrm{sign}\left(r_{i,k}\right)\cdot {\left(r_{i,k}\right)}^{2}$$

where $\mathrm{sign}\left(\cdot \right)$ denotes the signum function and is used to reflect discriminative information from the negative value of $r_{i,k}$. Then, the target frequency of each test signal is identified by the aforementioned Equation (4).

#### 2.2.3. Standard Task-Related Component Analysis

In TRCA, maximizing reproducibility between time-locked task trials leads to the spatial filters, which are capable of reflecting task-specific components. Assume SSVEP EEG signals of l-th trial $X^{\left(t\right)}\in {\mathbb{R}}^{N_{c}\times N_{s}},t=1,\;2,\;\cdots ,\;N_{t}$, where $N_{t}$ is the number of trials. Then, a linear combination of $X^{\left(t\right)}$ is written as $Y^{\left(t\right)}=w^{T}X^{\left(t\right)}$.

The TRCA method aims at designing the weight vector w which is obtained by maximizing the sum of covariance between available combinations of all trials. The covariance between $t_{1}$-th and $t_{2}$-th trials are computed as

(7) $$\overset{N_{t}}{\underset{\begin{array}{c} t_{1}\;,\;t_{2}=1\\ t_{1}\;\neq t_{2} \end{array}}{\sum }}\mathrm{Cov}\left(Y^{\left(t_{1}\right)}\;,Y^{\left(t_{2}\right)}\right)\;\;\;=\;\overset{N_{t}}{\underset{\begin{array}{c} t_{1}\;,\;t_{2}=1\\ t_{1}\;\neq t_{2} \end{array}}{\sum }}\overset{N_{c}}{\underset{c_{1}\;,c_{2}=1}{\sum }}w_{c_{1}}w_{c_{2}}\mathrm{Cov}\left(X_{C_{1}}^{\left(t_{1}\right)}\;,X_{C_{2}}^{\left(t_{2}\right)}\right)\;=\;\;w^{T}Sw$$

where Cov(· ,·) denotes the covariance between two variables, $X_{C_{1}}^{\left(t_{1}\right)}$ and $X_{C_{2}}^{\left(t_{2}\right)}$ denote $C_{1}$-th and $C_{2}$-th channels of EEG signals of $X^{\left(t_{1}\right)}$ and $X^{\left(t_{2}\right)}$, respectively.

Denote a concatenated matrix of all trials $X^{\left(t\right)}$ as $\hat{X}=\left[X^{\left(1\right)}\;X^{\left(2\right)}\;\ldots \;X^{\left(N_{t}\right)}\right]$. Constraining the variance of $Y^{\left(t\right)}$ by normalizing to one leads to

(8) $$\mathrm{Var}\left(Y^{\left(t\right)}\right)=\;\overset{N_{c}}{\underset{c1\;,\;\;c2=1}{\sum }}w_{c_{1}}w_{c_{2}}\mathrm{Cov}\left({\hat{X}}_{C_{1}}\;,{\hat{X}}_{C_{2}}\right)\;\;\;\;\;\;\;=w^{T}Qw\;\;\;\;\;\;\;=1$$

where Var(·) denotes the variance of a variable, ${\hat{X}}_{C_{1}}$ and ${\hat{X}}_{C_{2}}$ denote the $C_{1}$-th and $C_{2}$-th channels of $\hat{X}$, respectively.

Finally, the optimal weight vector $\hat{w}$ can be obtained through a constrained optimization problem as follows:

(9) $$\hat{w}=\;\arg\;\underset{w}{\max}\frac{w^{T}Sw}{w^{T}Qw}$$

In Equation (9), the optimal weight vector $\hat{w}$ is the eigenvector of $Q^{-1}S$, which corresponds to the largest eigenvalue. In the SSVEP-based BCI, a spatial filter approach such as TRCA has the effect of eliminating background activities by filtering out the principal components inherent in EEG signals [17,18]. In TRCA, for each target frequency, the correlation coefficient between the test signal and the individual template is determined from the training signal with the given optimal spatial filters as follows:

(10) $${\hat{\rho }}_{i}=\;\rho \left(w_{i}^{T}\bar{X}\;,w_{i}^{T}Y_{i}\right),\;\;i=1,2,\;\cdots ,\;N_{f}$$

Subsequently, target identification is performed as follows:

(11) $$f_{target}=\;\underset{i}{\max}{\hat{\rho }}_{i},\;\;i=1,\;2,\;\cdots ,\;N_{f}$$

### 2.3. The Proposed Two-Step TRCA

As mentioned earlier, advanced versions based on the spatial filter accomplished improved performance of frequency identification of SSVEP. In [16,18], using an ensemble approach yielded better robustness and superior performance than standard TRCA by integrating the spatial filters of all target frequency.

**Figure 1:**
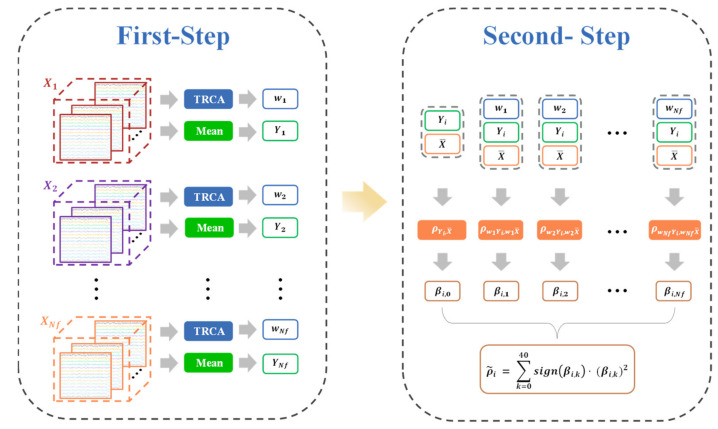
Flowchart of the proposed TSTRCA method. In the first step, the standard TRCA is employed to compute the spatial filters for each target frequency in the training data $X_{i}\in \;{\mathbb{R}}^{N_{c}\times N_{s}\times N_{t}},\;i=1,\;2,\;\cdots ,\;N_{f}$ and obtain the individual templates, i.e., $Y_{i},\;i=1,\;2,\;\cdots ,\;N_{f}$, by group averaging across multiple training blocks. Here, the remaining blocks are stored as test data. Followingly, in the second step, the obtained spatial filter $w_{i}$ for each target frequency is used in yielding the correlation coefficients between the test data $\bar{X}$ and the individual template $Y_{i}$. We repeat this procedure for test data and all individual templates to compute the parameters $\beta_{i,\;k},\;k=1,\;2,\;\cdots ,\;N_{f}$. $\beta_{i,\;0}$ denotes the correlation coefficient without the spatial filter.

With this regard, we propose a novel SSVEP frequency recognition method by utilizing the relationship between all spatial filters and individual templates, which is referred to as two-step TRCA (TSTRCA). Figure 1 shows the flowchart of the proposed method. The proposed TSTRCA method consists of two steps: (1) First-step: construction of the spatial filters using standard TRCA and individual templates by averaging SSVEP EEG signals except test data corresponding to the target frequencies; (2) Second-step: target identification based on an ensemble approach. 

The first step aims to obtain spatial filters and individual templates from training data for each target frequency as done in standard TRCA. In the second step, we emphasize the relationships between the test data and individual template to yield the informative features for frequency recognition. 

We newly formulate the parameter $\beta_{i,\;k}$ to further intensify the correlation coefficient with the feature corresponding to target frequency by redefining the relationship between test data and individual template. Specifically, the parameter $\beta_{i,\;k}$, *k* $=1,\;2,\;\cdots ,\;N_{f}$ is defined as the correlation coefficient between the i-th individual template and the spatial filter for the k-th target frequency with the test data, which is given by

(12) $$\beta_{i\;}\;=\;\left[\begin{array}{c} \beta_{i,0}\\ \beta_{i,\;\;1}\\ \beta_{i,\;\;2}\\ \vdots \\ \beta_{i,\;\;N_{f}} \end{array}\right]\;=\;\left[\begin{array}{c} \rho \left(Y_{i},\bar{X}\;\right)\\ \rho \left(w_{1}Y_{i},\;w_{1}\bar{X}\right)\\ \rho \left(w_{2}Y_{i},\;w_{2}\bar{X}\right)\\ \vdots \\ \rho \left(w_{N_{f}}Y_{i},\;w_{N_{f}}\bar{X}\right) \end{array}\right]$$

where $\beta_{i}$ is the correlation vector which consists of $\beta_{i,\;k},$ *k*$=1,\;2,\;\cdots ,\;N_{f}$, and $\beta_{i,0}$ denotes the correlation coefficient between $Y_{i}$ and $\bar{X}$ without a spatial filter. Then, the correlation coefficient ${\tilde{\rho }}_{i}$ is obtained as a weighted sum of squares of $\beta_{i,\;k}$ as an ensemble.

(13) $${\tilde{\rho }}_{i}=\;\overset{N_{f}}{\underset{k=0}{\sum }}\mathrm{sign}\left(\beta_{i,\;\;k}\right)\cdot {\left(\beta_{i,\;\;k}\right)}^{2}$$

where $\mathrm{sign}\left(\cdot \right)$ is used to reflect discriminative information from the negative value of $\beta_{i,\;k}$.

Finally, the target identification of the proposed TSTRCA method is calculated as follows:

(14) $$f_{target}=\;\underset{i}{\max}{\tilde{\rho }}_{i},\;\;i=1,\;2,\;\cdots ,\;N_{f}$$

### 2.4. Frequency Recognition Based on Filter Bank Approach

Recently, the filter bank approach, which extracts independent components by decomposing the frequency band of the input signal into multiple sub-bands using band-pass filters, has considerably contributed to improving the classification performance of BCI models [23,24]. For instance, the filter bank common spatial pattern (FBCSP) integrated the filter bank and the standard CSP, thus yielding an improved classification accuracy by correctly extracting the frequency bands that have prominently feature in the motor imagery BCI [23]. Similarly, the filter bank CCA (FBCCA) provided an improved frequency recognition performance compared to the conventional CCA [24]. Inspired by these results, we further adopted the filter bank approach to the proposed TSTRCA and compared the standard TRCA with the filter bank.

As introduced in [18], the filter bank approach in SSVEP-based BCI can effectively separate sub-band components, including independent information embedded in the harmonic frequency bands. In [24], depending on the type of sub-band components, the filter bank approach consists of three categories. Here, we use the third one, which is referred to as the M_3_ method. By using the M_3_ method, we can obtain multiple harmonic frequency bands with a high cut-off frequency. In the M_3_ method, the cut-off frequency range of sub-band is set between b×8 Hz and 90 Hz, where b indicates the sub-band index. The zero-phase Chebyshev Type Ⅰ infinite impulse response (IIR) is used as a band-pass filter. After that, the arranged bth sub-band is applied to SSVEP EEG signals and learned spatial filters for each target frequency to generate a set of correlation vectors between test data and individual template. Finally, in order to recognize the target frequency, a set of correlation vectors are combined into a single metric using the linear combination method presented by [25] and is given by

(15) $$\rho_{i}=\;s\left(b\right)\cdot \left(r_{i}^{b}\right),\;\;i=1,\;2,\;\cdots ,\;N_{f}$$

where $r_{i}^{b}$ is a set of correlation vectors according to bth sub-band and $s\left(b\right)=\;b^{\left(-1.25\right)}+0.25$. Here, $s\left(b\right)$ plays a role in compensating for the reduction in the SNR of SSVEP harmonic as the response frequency increases [24]. Then, the target identification is carried out using a given Equation (14).

## 3. Results

### 3.1. Performance Evaluation

This work was performed in the MATLAB environment on an Intel 3.60 GHz Core i7 with 64GB of RAM. In addition, we used the MATLAB codes, such as TRCA and filter bank method provided on the website (https://github.com/mnakanishi/TRCA-SSVEP (accessed on 12 February 2021)).

To evaluate the proposed TSTRCA method compared to the existing SSVEP frequency recognition methods such as CCA, ExtCCA, and TRCA, we used the classification accuracy and the information transfer rate (ITR) as two metrics to measure the frequency detection performance.

The ITR is described as the amount of information transmitted by a system’s output and given by [26]

(16) $$\mathrm{ITR}\;=\;\left(\log_{2}N_{f}+P\log_{2}P+\left(1-P\right)\log_{2}\left[\frac{1-P}{N_{f}-1}\right]\right)\times \left(\frac{60}{T}\right)$$

where $N_{f}$ indicates the number of target frequency, P is the classification accuracy, and T is the selected TW for visual stimulation including gaze shifting time. In this work, we predetermined the gaze shifting time of 0.5 s as presented in [20] and evaluated ITR for TWs with an interval of 0.1 s from 0.2 to 1.0 s. In addition, we used one-way repeated measure ANOVA as a statistical analysis to determine the significant difference.

A leave-one-out cross validation was applied to estimate the performance of various SSVEP frequency recognition methods for test data. Among the six trials in SSVEP EEG signals as described in Section 2.1, the five trials were comprised of the training data and the remaining trial was used as the test data. This process was repeated six times and the average values of all six accuracies and ITRs were represented as an average accuracy and ITR corresponding to the target frequency.

### 3.2. Target Identification Performance

**Figure 2:**
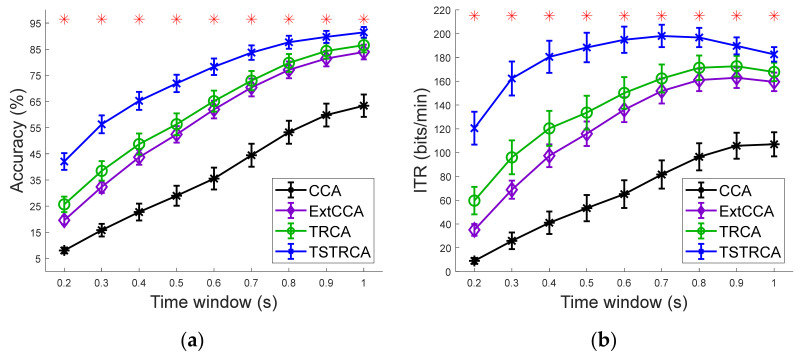
Average results of four methods without filter bank. (**a**) Average accuracy and (**b**) simulated ITR across 35 subjects for different time windows (TWs). Error bars represent standard errors. The asterisks indicate significant difference between four methods obtained by one-way repeated measures ANOVA (*p* < 0.001).

Figure 2a,b show the average accuracies and simulated ITR of CCA, ExtCCA, TRCA, and the proposed TSTRCA across all subjects for different TWs, respectively. Among 64 channels of EEG signals, we used 9 channels (Pz, PO5, PO3, POz, PO4, PO6, O1, Oz, O2) as in [11]. As can be seen in these figures, the standard TRCA was superior to CCA and ExtCCA and the proposed TSTRCA showed the best performance compared with other methods in terms of both average accuracy and ITR. Note that, for the short TWs from 0.2 to 0.5 s, the superiority of the proposed TSTRCA was clearer than the cases of TWs above 0.6 s. For example, the differences of average accuracy between TRCA and TSTRCA for the time window of 0.2 and 1.0 s are approximately 17% and 3%, respectively. In the figures, one-way repeated measures ANOVA analysis indicated that there were significant difference between four methods across all TWs in terms of average accuracy (TW = 0.2: F(3, 102) = 35.46, *p* < 0.001; TW = 0.3: F(3, 102) = 30.55, *p* < 0.001; TW = 0.4: F(3, 102) = 26.34, *p* < 0.001; TW = 0.5: F(3, 102) = 23.79, *p* < 0.001; TW = 0.6: F(3, 102) = 23.42, *p* < 0.001; TW = 0.7: F(3, 102) = 21.28, *p* < 0.001; TW = 0.8: F(3, 102) = 18.6, *p* < 0.001; TW = 0.9: F(3, 102) = 16.44, *p* < 0.001; and TW = 1.0: F(3, 102) = 16.13, *p* < 0.001). In addition, for simulated ITR, significant difference between four methods were similarly observed (TW = 0.2: F(3, 102) = 25.56, *p* < 0.001; TW = 0.3: F(3, 102) = 25.42, *p* < 0.001; TW = 0.4: F(3, 102) = 22.98, *p* < 0.001; TW = 0.5: F(3, 102) = 21.25, *p* < 0.001; TW = 0.6: F(3, 102) = 21.3, *p* < 0.001; TW = 0.7: F(3, 102) = 19.92, *p* < 0.001; TW = 0.8: F(3, 102) = 18.68, *p* < 0.001; TW = 0.9: F(3, 102) = 16.59, *p* < 0.001; and TW = 1.0: F(3, 102) = 16.65, *p* < 0.001).

Table 1 summarizes the statistical analysis results of the performance of each method between the different number of channels. ExtCCA, TRCA, and TSTRCA showed statistical difference as the number of channels increased. In addition, Table 2 exhibits the statistical analysis results of the performance between four methods when a different number of channels was used. It can be observed that except for the channels of 3, there was a statistically significant difference between the four methods.

**Figure 3:**
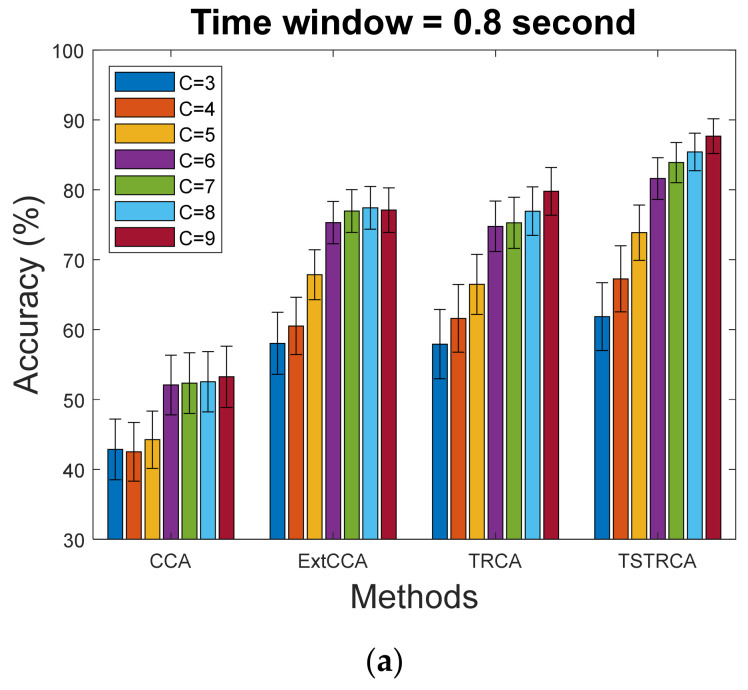

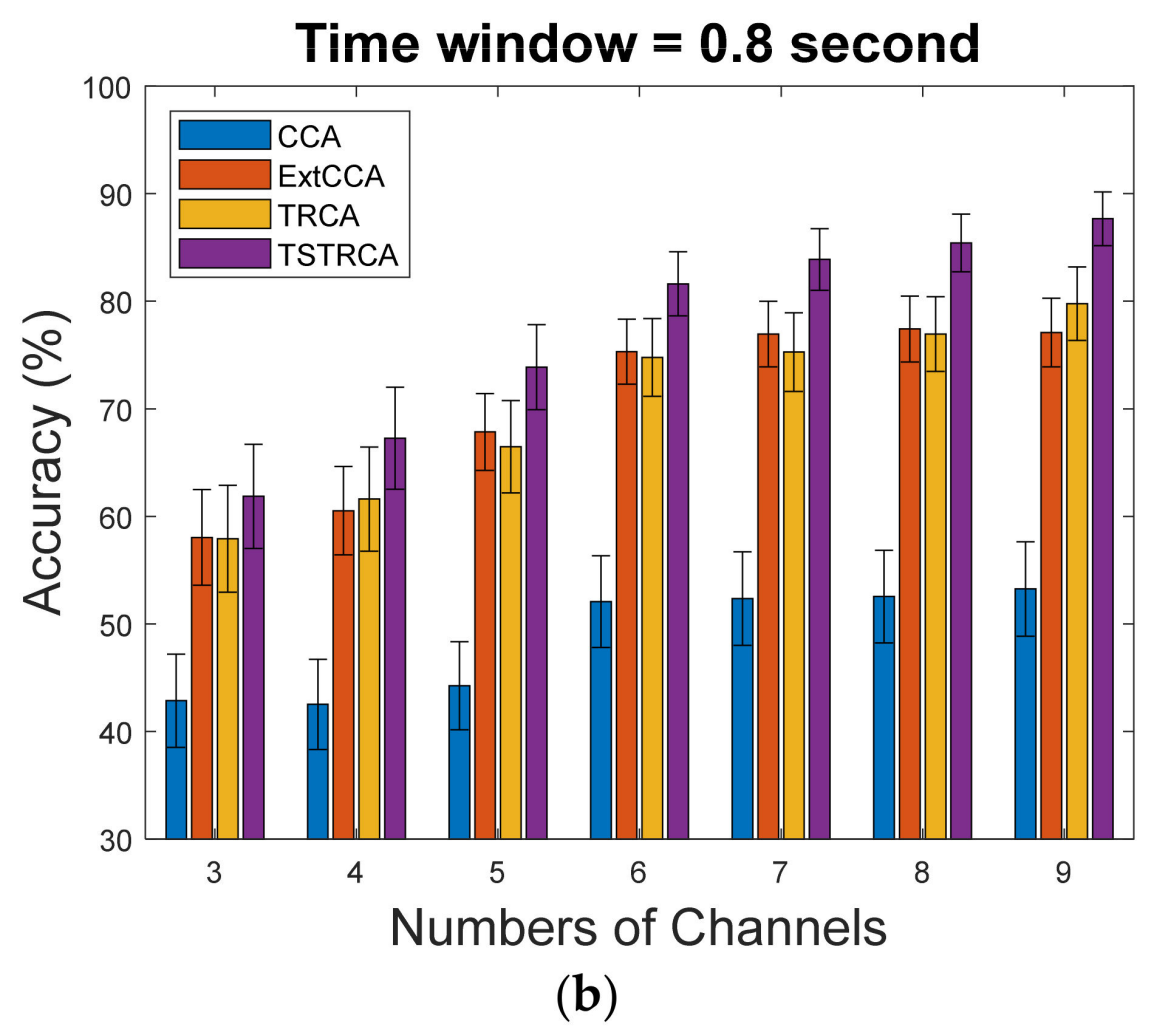
Average accuracy across 35 subjects for four methods in cases of the different number of channels with TW of 0.8 s. (**a**) Methods; (**b**) The number of channels. Error bars represent standard errors.

To further verify the performance comparison of the four methods, we examined the average performance for the number of channels. Here, the TW was set to 0.8 s. Figure 3 illustrates the average accuracy of the four methods in cases where a different number of channels was used. In Figure 3a, we can observe that the average accuracy increased as more channels were used for all methods. The average accuracy comparison results for all methods in terms of the number of channels are shown in Figure 3b. As can be seen in the figure, across all cases of the number of channels, the proposed TSTRCA achieved the best average accuracy among the four methods. Note that the average accuracy of TSTRCA with a lower number of channels was comparable to ExCCA and TRCA or outperformed CCA, ExCCA, and TRCA.

**Figure 4:**
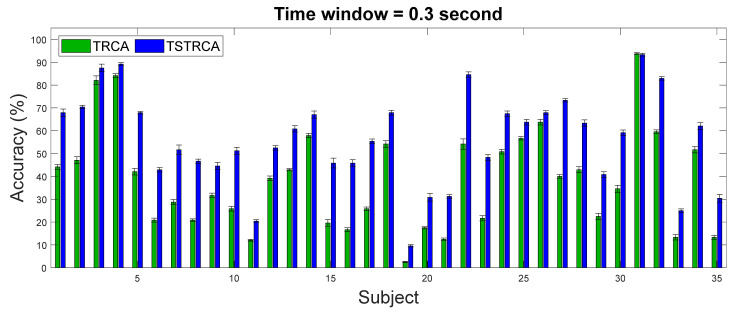
Average accuracy across all subjects for TW of 0.3 s using TRCA and TSTRCA. The error bars represent standard errors. Green and blue bars represent the average accuracies of TRCA and TSTRCA, respectively.

**Figure 5:**
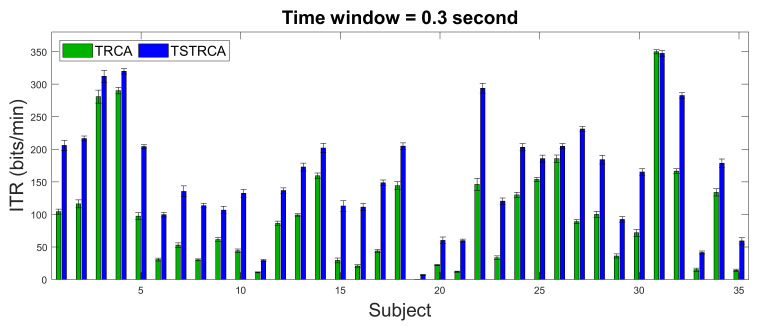
Average ITR across all subjects for TW of 0.3 s using TRCA and TSTRCA. The error bars represent standard errors. Green and blue bars represent the average ITRs of TRCA and TSTRCA, respectively.

**Figure 6:**
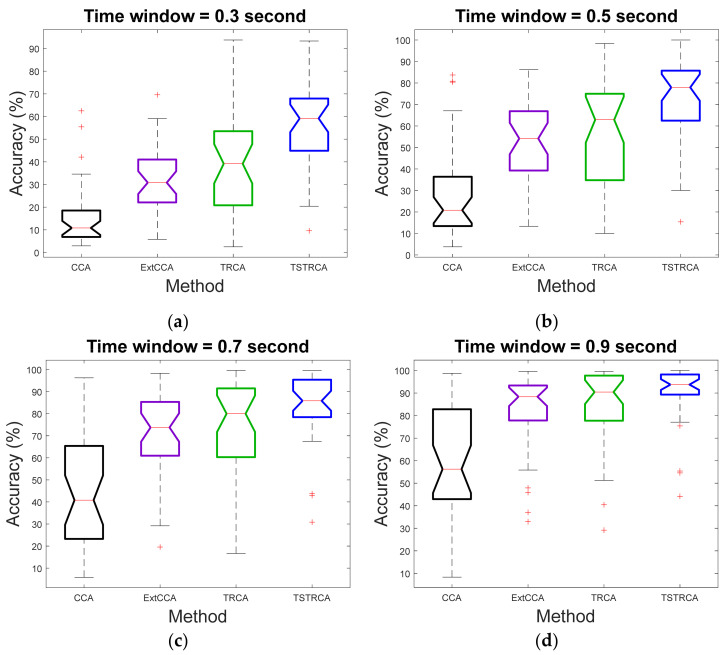
Median accuracy for TWs with an interval of 0.2 s from 0.3 s to 0.9 s. (**a**) 0.3 s time window. (**b**) 0.5 s time window. (**c**) 0.7 s time window. (**d**) 0.9 s time window. Here, on each box, the central red line indicates the median, and the bottom and top edges of the box refer to the 25th and 75th percentiles, respectively. Whiskers extend to the maximum or minimum performance not considered by outliers, and outliers are denoted by ‘**+**’.

**Figure 7:**
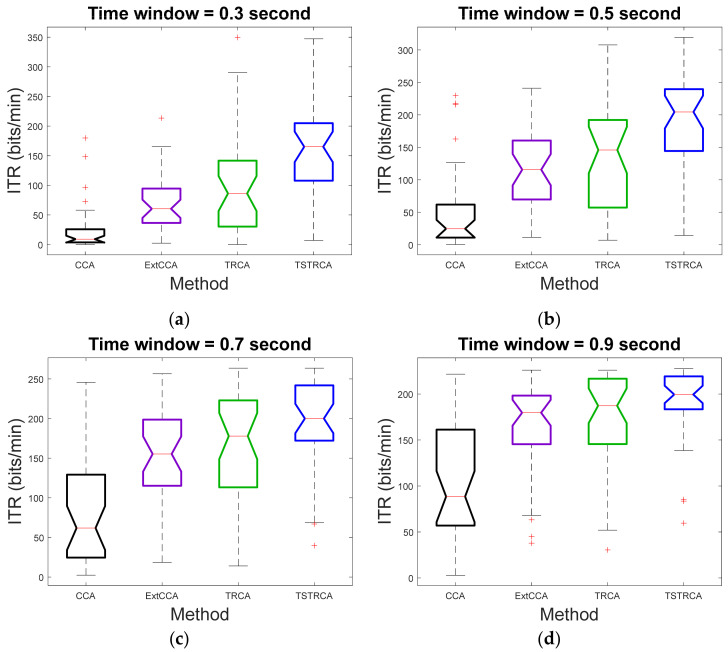
Median ITR for TWs with an interval of 0.2 s from 0.3 s to 0.9 s. (**a**) 0.3 s time window. (**b**) 0.5 s time window. (**c**) 0.7 s time window. (**d**) 0.9 s time window. Here, on each box, the central red line indicates the median, and the bottom and top edges of the box refer to the 25th and 75th percentiles, respectively. Whiskers extend to the maximum or minimum performance not considered by outliers, and outliers are denoted by ‘**+**’.

Figure 4 and Figure 5 illustrate the average accuracy and ITR of two methods—TRCA and TSTRCA—across all subjects for a TW of 0.3 s. As shown in the figures, the average accuracy and ITR were improved for most subject. However, the amount of improvement of accuracy can be biased by several subjects. Thus, to avoid the impact of a specific subject on average performance in the comparison analysis, we further investigated median accuracy and ITR analysis, as depicted in Figure 6 and Figure 7, respectively.

In Figure 6 and Figure 7, we verified that the proposed TSTRCA outperformed other methods in terms of median accuracy and median ITR for TWs with an interval of 0.2 s from 0.3 to 0.9 s. It implies that the performance improvement of TSTRCA came from improvement on most subjects, not due to specific subjects.

**Figure 8:**
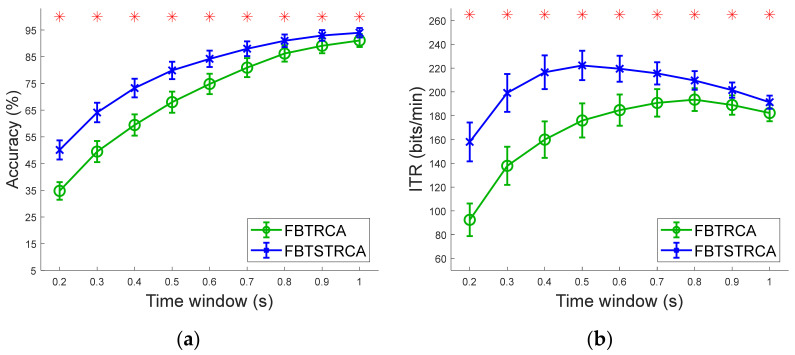
Average results of TRCA and TSTRCA with filter bank. (**a**) Averaged accuracy and (**b**) simulated ITR across 35 subjects for different TWs. Error bars represent standard errors. The asterisks indicate significant difference between four methods obtained by one-way repeated measures ANOVA (*p* < 0.001).

Finally, to validate the effect of the filter bank approach, we examined the performance of TRCA and the proposed TSTRCA with filter banks at different TWs. Figure 8a,b indicate the average accuracy and simulated ITR of TRCA and TSTRCA with a filter bank, which are referred to as FBTRCA and FBTSTRCA, respectively. As in TRCA and TSTRCA without a filter bank in Figure 2, we observed that the TSTRCA with a filter bank—FBTSTRCA—was superior to the TRCA with a filter bank—FBTRCA—across all TWs. Through one-way repeated measure ANONA analysis, we confirmed a significant difference between the two methods.

## 4. Discussions

Due to simplicity and improved performance, the standard CCA and its variants, such as L1-MCCA and MsetCCA, have contributed to enhanced SSVEP-BCI. Followingly, the use of spatial filters in SSVEP-BCI research has enhanced the performance of target frequency recognition significantly [18,25]. 

Recently, the TRCA approach, which extracts the spatial filers with task-specific components, has yielded notable improvement compared to conventional SSVEP-BCI methods [18,27]. However, the TRCA approach is beneficial especially for a sufficiently long length of SSVEP EEG recordings. In this regard, the proposed TSTRCA has provided improved performance for the recognition of target frequencies for short and long time windows.

The proposed TSTRCA consists of two steps. First, it aims to develop the spatial filters and individual templates using training data. Second, the target frequencies are identified by applying an ensemble classifier. In the second step, all spatial filters are utilized to accentuate the features corresponding to the target frequencies. The first and second steps correspond to the training stage and test stage of TRCA, respectively. In Figure 2, Figure 6, and Figure 7 of Section 4, the results demonstrate that the proposed TSTRCA yields enhanced accuracy and ITR compared to conventional SSVEP methods. While ExtCCA utilizes two reference signals, TSTRCA uses a single reference, thus implying its simplicity.

Furthermore, we carried out a performance comparison of the proposed TSTRCA and conventional SSVEP methods in terms of precision, recall, and F1-score, shown in Table 3. These metrics are obtained by averaging each metric over all subjects and all trials with a TW of 0.5 s. As can be seen, the TSTRCA shows more robust performance than other methods in short time windows. 

Recently, several fusion-based SSVEP-BCI studies have shown the remarkable performance of frequency recognition. Liu et al. [19] developed FoCCA, which fuses all correlation coefficients of the standard CCA. While FoCCA represented approximately 80% accuracy with a TW of 2 s, the proposed TSTRCA shows an 83.71% accuracy with a TW of 0.7 s. Besides, the average accuracy and simulated ITR of fusion of maximum signal fraction analysis (FoMSFA) [28] were less than 31% and 100 bits/min for a TW of 0.2 s, respectively. Compared to these results, TSTRCA demonstrates 42.06% accuracy and 120.51 bits/min ITR using the same length of TW. This comparison suggests the superiority of TSTRCA over fusion-based SSVEP-BCI methods.

For practical use, the improved performance of the proposed TSTRCA with a short time window suggests its promising capability as a new communication tool for both healthy and disabled people. Thus, SSVEP EEG signals would play a role in daily life, such as the use of photoplethysmography (PPG) and electrocardiogram (ECG) [29,30,31]. 

The proposed TSTRCA was designed and evaluated on the offline experiment. Thus, future works should be conducted to (1) establish the real-time SSVEP-BCI system using TSTRCA, (2) explore how different spatial filtering mechanisms address the trade-off between computational complexity and the performance for SSVEP frequency recognition, and (3) construct the SSVEP-BCI EEG dataset extracted from the various subjects and environments to pursue a general-purpose SSVEP-BCI framework by extending our work.

## 5. Conclusions

We presented a novel frequency recognition method for SSVEP-based BCI based on the TRCA method. The proposed TSTRCA accentuates the features corresponding to target frequencies by: (1) redefining a correlation vector based on the spatial filters of all target frequencies, (2) emphasizing the relationship between test data and individual templates using an ensemble classifier. Through validation using the SSVEP benchmark dataset, we confirmed that the proposed TSTRCA outperforms the existing SSVEP frequency recognition methods including the standard TRCA in terms of average accuracy and simulated ITR. Furthermore, we introduced the proposed TSTRCA with a filter bank, which is called FBTSTRCA, and validated its superior performance over the standard TRCA with a filter bank. The experimental results suggest that the proposed TSTRCA can play an important role for SSVEP frequency recognition since it possesses efficacy in frequency recognition in case of a short time window. These properties of TSTRCA imply the suitability as a promising frequency recognition strategy for SSVEP-based BCI applications.

## Figures and Tables

**Table 1:** Statistical analysis results of average accuracy and ITR between the different number of channels for each method.

		Method			
		CCA	ExtCCA	TRCA	TSTRCA
**Accuracy**	F(6, 204)	1.39	5.59	4.22	7.43
	*p*	0.22	<0.001	<0.001	<0.001
**ITR**	F(6, 204)	1.29	5.57	4.1	7.74
	*p*	0.26	<0.001	<0.001	<0.001

**Table 2:** Statistical analysis results of average accuracy and ITR between four methods for the different number of channels.

		Channels						
		3	4	5	6	7	8	9
**Accuracy**	F(3, 102)	3.26	5.73	10.6	13.63	15.07	17.2	18.6
	*p*	0.002	0.001	<0.001	<0.001	<0.001	<0.001	<0.001
**ITR**	F(3, 102)	3.66	6.21	10.55	12.8	14.53	16.83	18.68
	*p*	0.001	<0.001	<0.001	<0.001	<0.001	<0.001	<0.001

**Table 3:** Comparison of performance (Precision, Recall, and F1-score) of SSVEP frequency recognition for TW of 0.5 s.

	Method (Average ± std. dev. in %)			
	CCA	ExtCCA	TRCA	TSTRCA
**Precision**	22.19 ± 21.63	42.73 ± 19.86	48.57 ± 25.62	64.33 ± 22.81
**Recall**	28.94 ± 23.24	52.43 ± 19.84	56.32 ± 24.89	71.92 ± 20.59
**F1-score**	25.14 ± 22.45	46.94 ± 20.02	52 ± 25.46	67.78 ± 21.94

## Data Availability

No new data were created or analyzed in this study. Data sharing is not applicable to this article.
